# Change in carbohydrate and enzymes from harvest to sprouting in garlic

**DOI:** 10.1002/fsn3.299

**Published:** 2015-11-01

**Authors:** Kambiz Mashayekhi, Siamak Mohammadi Chiane, Manizheh Mianabadi, Farshid Ghaderifar, Seyyed Javad Mousavizadeh

**Affiliations:** ^1^ Department of Horticultural ScienceGorgan University of Agricultural Sciences and Natural ResourcesGorganIran; ^2^ Department of BiologyFaculty of SciencesUniversity of GolestanGorganIran; ^3^ Department of AgronomyGorgan University of Agricultural Sciences and Natural ResourcesGorganIran

**Keywords:** Carbohydrate, chlorophyll, enzyme, sprouting, storage

## Abstract

Changes in carbohydrates, enzymes, and pigments were investigated in the Red Garlic (*Allium sativum* L.) cv. Azarshahr bulbs in storage from harvest to sprouting. For storage period, garlic cloves of the same diameter with 3–4 g weight were arranged in dark condition and exposed to 4 and 21°C, separately. Soluble sugar, total sugar, glucose, sucrose, fructose, starch, chlorophyll *a*,* b*,* ab*, carotenoid, anthocyanin, lipase, *α*‐amylase, and protease were measured every 2 weeks up to sprouting time. Result revealed that starch, lipase, and protease levels declined at the end of storage when clove sprouting started at both 4 and 21°C storage temperature. Starch, glucose, chlorophyll *a*,* b*,* ab*, and carotenoid content during the first 14 days and sucrose at 42 days showed a decreasing trend. Anthocyanin showed an increasing trend 14 and 42 days after harvesting and then decreased at the end of storage (when sprouting began) at both 4 and 21°C storage periods. Finally, starch, glucose, and sucrose measurement can be used as a criterion to predict sprouting time of garlic, due to the decrease in the levels of starch, lipase, and protease, and increase in the levels of *α*‐amylase, glucose, and sucrose in garlic cloves under storage.

## Introduction

Garlic (*Allium sativum* L.), a plant of the Liliaceae family, plays an important role in human nutrition and has medicinal value (Rubatzky and Yamaguchi 1997). Plants store sugars usually as large molecules of starch which are required to produce energy; plants are required to break them into smaller molecules (Hapkins 1999).

Previous reports indicated that garlic dormancy ends by storage at low temperatures (Cantwell et al. 2003 and Vazquez‐Barrios et al. 2006). Arguello et al. (2001) reported that cold treatment at 4°C and Gibberellic Acid (GA3) initiated the garlic sprouting process. During cold storage, due to hydrolysis of starch, carbohydrates in onion tissues acquire mobility (Fulton et al. 2001). Carbohydrate macromolecules such as starch are converted into simple sugars such as sucrose, glucose, and fructose, making this process possible. Transport and accumulation of these substances (sugars) that are source of energy, are used in cellular metabolism, and produce the energy needed for plant growth (Langens‐Grrits et al. 2003). In addition to energy transfer in plants, carbohydrates are also involved in the regulation of gene expression (Gupta and Kaur 2005; Iraqi et al. 2005).

Despite the large‐scale production of garlic in Iran, research into the changes in garlic carbohydrates from harvest to sprouting is scarce. We believe the changes in garlic carbohydrates play an important role from dormancy to sprouting. This study aimed to understand the physiological changes in carbohydrates of garlic from dormancy to sprouting. Understanding these changes create optimal conditions for clove sprouting, plant growth, and ultimately increases in yield.

## Materials and Methods

### Plant materials

Red garlic (*A*. *sativum* L.) cv. Azarshahr bulbs were collected from local fields in Azarshahr when they were fully ripe and their leaves began browning. Four thousand bulbs with the same diameter were selected. Pieces of garlic were arranged in plastic trays enclosed in aluminum foils in order to avoid light.

### Storage condition and sprout treatment

**Figure 1:**
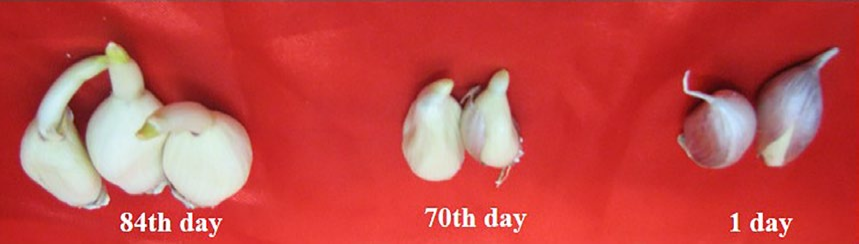
Sprouting of garlic bulbs from 1 day up to 84 days. Developed and greenish bud was considered as sprouting.

Garlic cloves of the same diameter and weighing 3–4 g were taken in plateaus and arranged in a dark enclosure to avoid light and treated in two ways: (1) at 21°C with 45% humidity (common condition) and (2) at 4°C with 60% humidity, the latter of which is the most suitable temperature for garlic sprouting (Hartman 2002). Sampling was carried out for 2 weeks up to sprouting. Bulbs were observed every 14 days for up to 84 days and were considered sprouting when the bud was developed and greenish (Fig. 1).

### Determination of traits

#### Soluble sugar content

Soluble sugar was determined according to the Omokolo et al. (1996) method by application of 80% ethanol.

#### Total sugar content

Total sugar was determined according to the Antron Method by spectrophotometry (S 2000 UV/Vis) at 620 nm (McCready et al. 1950).

#### Glucose content

Glucose content was calculated using dinitrosalicylic acid by spectrophotometry at 575 nm (Miller 1959).

#### Sucrose content

Sucrose content was determined according to the Antron Method by spectrophotometry at 620 nm (Handel 1968).

#### Fructose content

Fructose content was calculated by resorcinal Method by spectrophotometry at 520 nm (Ashwell 1957).

#### Starch measurement

Starch was determined according to the Anthrone Method by spectrophotometry at 620 nm (McCready et al. 1950).

#### Carotenoid and chlorophyll contents

Carotenoid and chlorophyll contents were extracted using acetone, from 1 g of garlic. The amount of these pigments was measured by Arnon (1956) method pectrophotometry (S 2000 UV/Vis) at 480, 510, 645, 652, and 663 nm, and expressed as mg/g fresh weight.

#### Anthocyanin content

Anthocyanin contents of garlic cloves were measured according to Wanger (1979). One gram of clove tissue was extracted via 10 mL methanol and incubated overnight at 4°C in the dark. The slurry was centrifuged (SIGMA‐3K30) at 4000 g for 10 min. The supernatant, which contained anthocyanin, was determined by pectrophotometry at 520 nm.

#### α‐Amylase activities

The amount of *α*‐amylase activities was measured by 3,5‐dinitro salicylic acid color indicator and starch substrate of 1%, spectrophotometry at 540 nm (Bernfeld 1955).

#### Lipase activities

Lipase activities was estimated according to Sadeghipour and Bhatla (2002). A suspension of urea‐washed garlic bodies treated with trypsin was taken in 0.1 mol/L Tris buffer (pH 7.5). Lipolysis was started by the addition of 20 μL of a garlic membrane preparation containing lipase. To 1 mL of benzene extract, 0.5 mL Rhodamine 6G reagent was added and the fatty acids released were quantified by reading the absorbance at 535 nm. A standard curve was made using palmitic acid. Lipase activity was expressed as μmol fatty acids released per minute/g FW.

#### Protease activities

Protease assay was determined by the Anson (1938) method. Anson unit is the amount of enzyme which, under the specified test conditions (2% casein as substrate, pH 7.0; for 15 min, at different temperatures analyzed) hydrolyzes casein at a speed that facilitates release, in 1 min, of hydrolysis products soluble in trichloroacetic acid; this provides coloration equivalent, measured at OD_670nm_, to 1 μmol of tyrosine, in the presence of the Folin‐Ciocalteu reagent by using a tyrosine standard curve over the range 0.02–0.24 μmol/mL.

### Statistical analysis

The experiment was established in a factorial (two levels of temperature and seven levels of storage time) layout based on completely randomized design with 12 replications. Data were analyzed statistically using analysis of variance in SAS 9.1 software (SAS Institute Inc., Cary, NC). The data of anthocyanin, carotenoid, chlorophyll *a*,* b,* and *ab* were normalized by root square ($\sqrt{X}$). Differences among the means were determined for significance at *P *<* *0.05 using Duncan test.

## Results and Discussions

**Figure 2:**
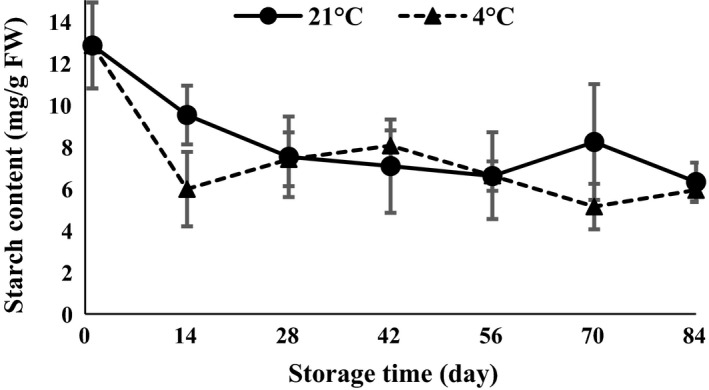
Starch content changes during storage period at 4 and 21°C.

The results of ANOVA (Table 1) demonstrated that starch was affected by temperature and time of storage (*P *<* *0.01). Results revealed that garlic cloves stored at 4 and 21°C showed a similar pattern of changes in carbohydrates and similar time for sprouting. Cantwell et al. (2003) suggested that storage of garlic at temperatures of 5–18°C had promoted garlic sprouting. In accordance with this result, in this study, starch decomposed gradually at 84 days of storage (Fig. 2). Lee (2007) demonstrated that the hydrolysis of starch at different temperatures is associated with physiological processes. Onion bud dormancy is broken by chilling treatment which causes changes in soluble sugars and oligosaccharides (Benkeblia and Selselet‐Attou 1999).

**Table 1:** ANOVA of garlic storage at 21 and 4°C during different times

Source of variance	df	Starch	Total sugar	Sucrose	Glucose	Fructose	Anthocyanin	Chlorophyll *a*
Temperature (*a*)	1	32.31**	3.44 ns	952.5 ns	675.2**	20.11*	0.0003^ns^	0.0009^ns^
Time (*b*)	6	124.29**	1555.2**	27,385.5**	7084.6**	27.42**	0.015**	0.18**
*a* × *b*	6	17.83**	166.2 ns	288.15 ns	187.9**	29.48**	0.003*	0.02**
Error	154	2.80	252.6	479.5	60.57	3.18	0.001	0.002
CV%		21.32	31.72	25.41	14.56	32.06	29.35	22.97

Source of variance	df	Chlorophyll *b*	Chlorophyll *ab*	Carotenoid	*α*‐Amylase	Lipase	Protease
Temperature (*a*)	1	0.033**	0.027*	0.78 ns	29.25 ns	36.90**	0.05**
Time (*b*)	6	0.329**	0.54**	22.57**	3017.3**	48.20**	0.52**
*a* × *b*	6	0.042**	0.048**	0.73**	28.5^ns^	26.86**	0.003^ns^
Error	154	0.004	0.006	0.23	26.73	0.46	0.004
CV%		26.12	24.19	34.43	24.27	18.40	20.09

**P <* *0.01, *P <* *0.05, ^ns^ *P *>* *0.05.

In the present study, starch content strongly declined 14 days after harvesting in both the 4 and 21°C storage periods (Fig. 2). Previous research indicated that the content of starch was the highest in the beginning of storage and reached its lowest value after chilling treatment at 4°C (Atashi et al. 2011).

**Figure 3:**
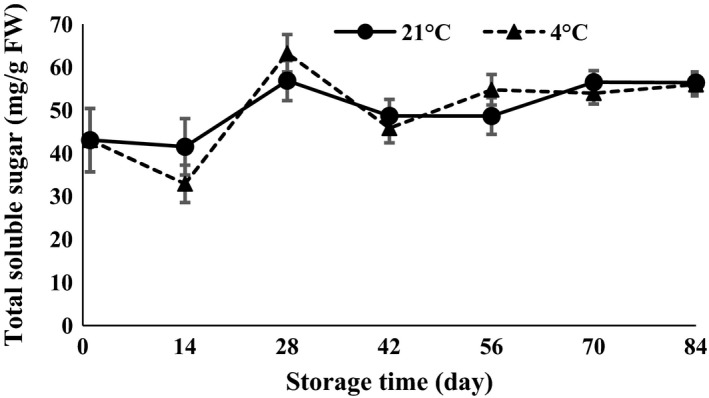
Total soluble sugar content changes during storage period at 4 and 21°C.

ANOVA (Table 1) showed that total soluble sugar (TSS) was statistically affected by time of storage (*P *<* *0.01). The TSS content decreased 14 days after storage and then increased at temperatures of 4 and 21°C (Fig. 3), indicating reduced sugar consumption in the metabolic pathway of the central bud of the clove. Vazquez‐Barrios et al. (2006) observed that storage of garlic at 5°C resulted in a 33% reduction in TSS.

**Figure 4:**
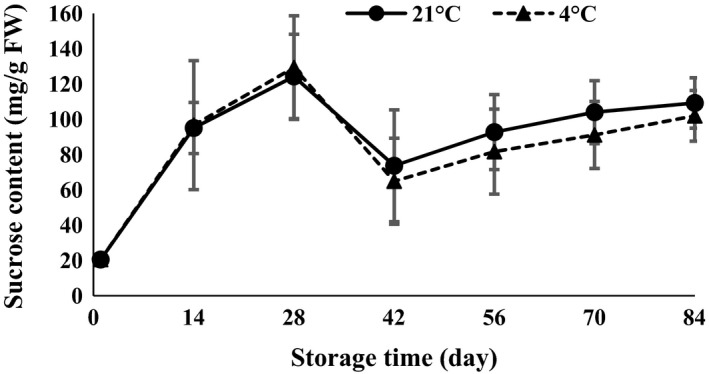
Sucrose content changes during storage period at 4 and 21°C.

The results of ANOVA (Table 1) revealed that sucrose was affected by time of storage (*P *<* *0.01). The results suggested that sucrose content strongly increased 28 days after storage at both 4 and 21°C (Fig. 4). Sucrose is the most important plant disaccharide and is the most common form of carbohydrate transported in plants (Hapkins 1999). Changes in the form of simple non‐structural carbohydrates (sucrose, glucose, and fructose) at the time of onion bulb formation demonstrated that glucose and sucrose had the highest and lowest increase, respectively (Kahane et al. 2001). Rosa et al. (2009) showed that starch content of *Chenopodium quinoa* exposed to low temperature (2.5°C) followed a decreasing trend on the second day, whereas sucrose content increased until Day 4 and then decreased until Day 9 of the experiment.

**Figure 5:**
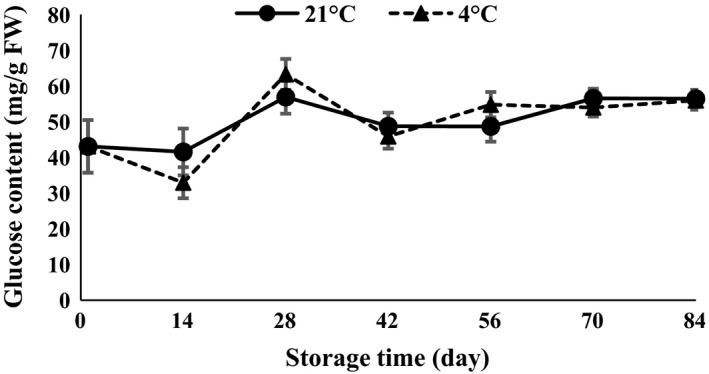
Glucose content changes during storage period at 4 and 21°C.

**Figure 6:**
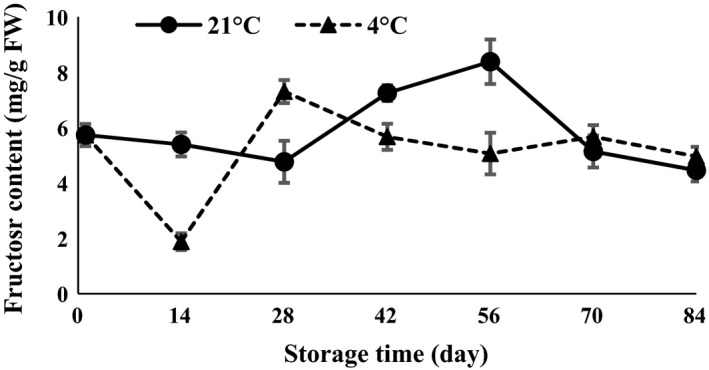
Fructose content changes during storage period at 4 and 21°C.

The results of ANOVA (Table 1) demonstrated that glucose and fructose were affected by temperature and time of storage (*P *<* *0.01). Glucose showed a decreasing trend in the first 14 days after harvesting and then increased toward the end of storage time at both 4 and 21°C (Fig. 5). On the other hand, the results demonstrated that fructose declined 28 and 14 days after the storage period at both 21 and 4°C, respectively (Fig. 6). Glucose content in the first 14 days (Fig. 5) and sucrose content at 42 days (Fig. 4) showed a decreasing trend due to growing of buds. The levels of fructose (Fig. 6) oscillated during germination. Each of the sugars plays a role in bud growth. Due to the increase or decrease in each of the sugars during storage, the amount of total soluble sugars (Fig. 4) also increases or decreases. During processes such as seed germination, relatively large amounts of simple sugars are produced (Hapkins 1999). Garlic microbulblets physiologically mature and are able to sprout after 90 days of storage (Arguello et al. 2001). Cloves exposed to 5°C for 15–30 days before sowing accelerate the maturity of bulbs compared with cloves at room temperature or 20°C (Rahim and Fordham 2001). Cold storage at 5°C for 5 weeks before planting enhanced the purple color of the garlic bulb (Dufoo‐Hurtado et al. 2013).

**Figure 7:**
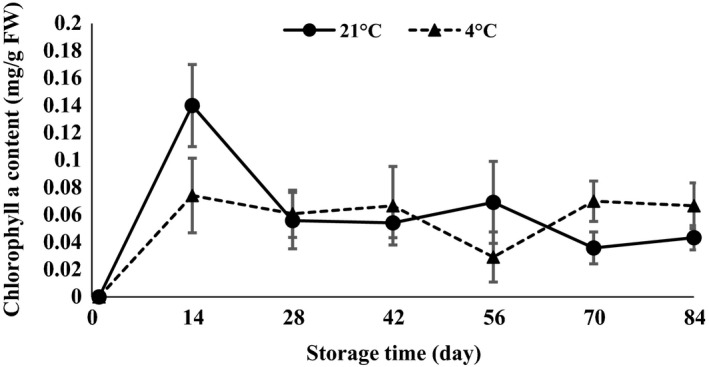
Chlorophyll *a* content changes during storage period at 4 and 21°C.

**Figure 8:**
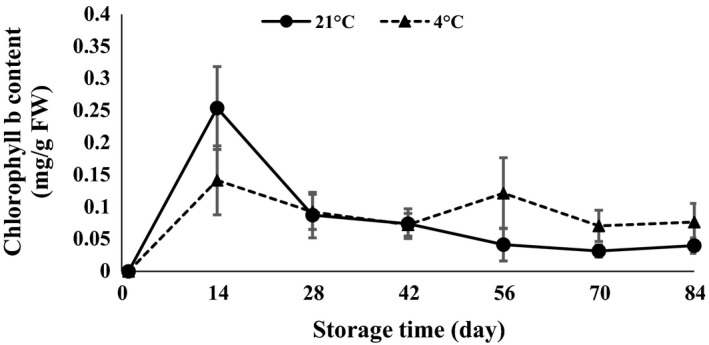
Chlorophyll *b* content changes during storage period at 4 and 21°C.

**Figure 9:**
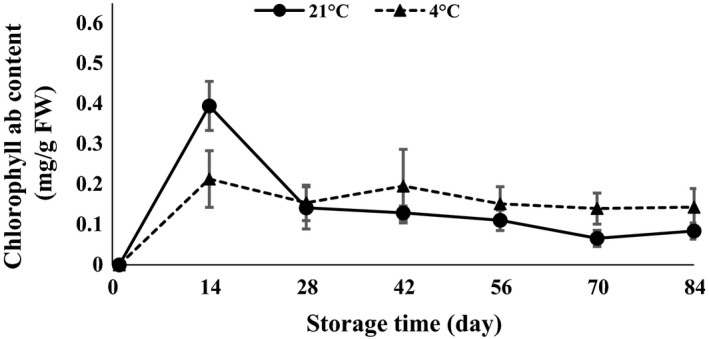
Chlorophyll *ab* content changes during storage period at 4 and 21°C.

**Figure 10:**
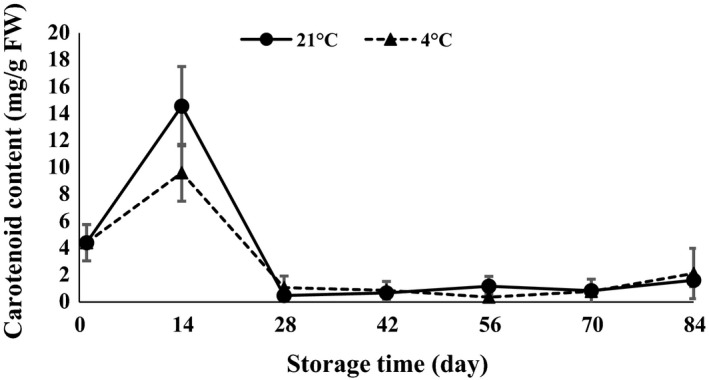
Carotenoid content changes during storage period at 4 and 21°C.

**Figure 11:**
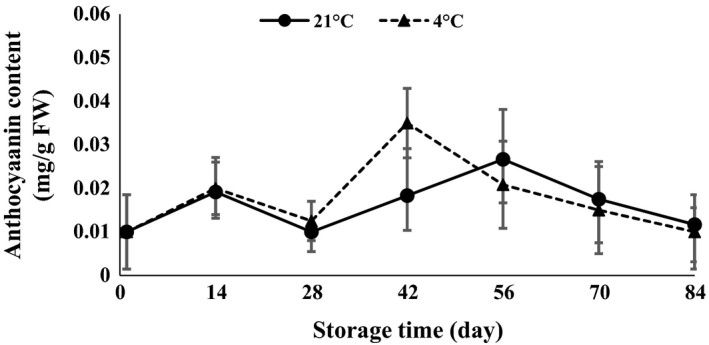
Anthocyanin content changes during storage period at 4 and 21°C.

ANOVA (Table 1) demonstrated that anthocyanin, carotenoid, chlorophyll *a*,* b*,* ab*, and lipase were statistically affected by interaction of temperature and time of storage. The results revealed that chlorophyll *a*,* b*,* ab,* and carotenoid content strongly increased in the first 14 days after storage and then decreased at both 4 and 21°C (Figs. 7, 8, 9, 10). The results showed that the content of chlorophyll *a*,* b,* and *ab* increased when glucose was decreased at day 14, which indicated that the glucose was used to produce chlorophyll. Atashi et al. (2011) reported that chlorophyll *a*,* b*,* ab*, and carotenoids exhibited the highest values after the 30‐day chilling period at 4°C. Anthocyanin showed an increasing trend 14 and 42 days after harvesting and then decreased toward the end of storage (when sprouting began) at both 4 and 21°C (Fig. 11).

**Figure 12:**
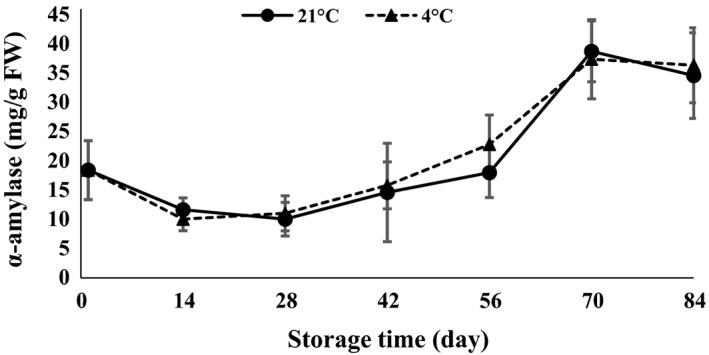
*α*‐Amylase content changes during storage period at 4 and 21°C.

**Figure 13:**
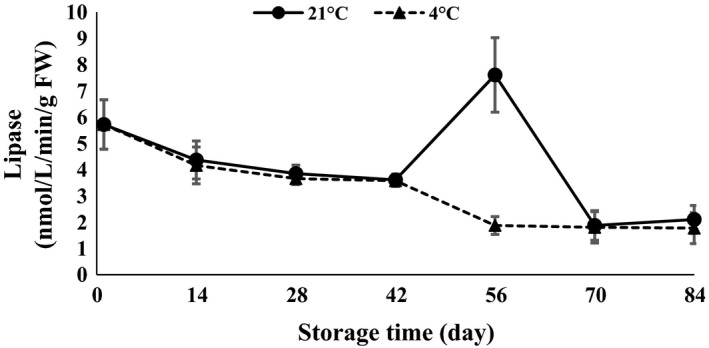
Lipase content changes during storage period at 4 and 21°C.

**Figure 14:**
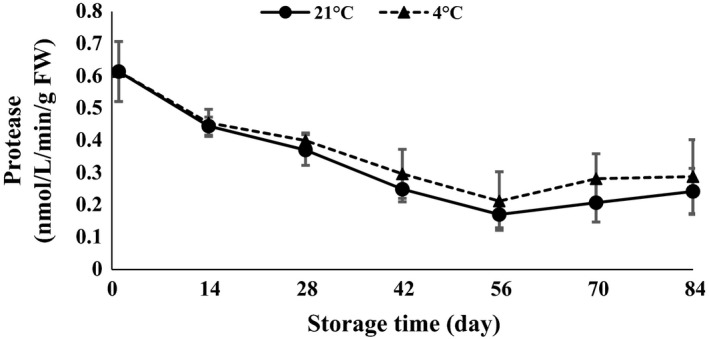
Protease content changes during storage period at 4 and 21°C.

*α*‐Amylase and protease were statistically affected by time of storage (*P *<* *0.01). *α*‐Amylase showed a decreasing trend in the first 14 days after storage and then increased toward the end of storage at both 4 and 21°C (Fig. 12). The activity of *α*‐amylase was increased in response to an increase in chilling period at 4°C (Atashi et al. 2011). Amylase activity in Ruditapes variegatus has been reported during storage temperature of 4–35°C by Lin et al. (2009). Lipase enzyme slowly decreased at 84 days of storage (Fig. 13). A sudden jump was observed 56 days after storage for 21°C storage. Levels of protease declined at the end of storage when clove sprouting started at both 4 and 21°C (Fig. 14). Arguello et al. (2001) stated that at the end of garlic dormancy, peaks of peroxidase activity were observed. During both cold stratification and warm incubation of walnut kernels, storage protein mobilization occurred in the cotyledon (Einali and Sadeghipour 2007).

## Conclusion

Some physiological changes occurred in garlic during storage time. The results of this experiment indicated that storage at both 4 and 21°C caused starch and sugar changes in garlic leading to sprouting. Some part of starch decomposed, leading to an increase in the content of glucose and sucrose in garlic tissue. When glucose increases, lipase and protease activity are reduced. However, starch, glucose, and sucrose are useful indices to predict sprouting time of garlic.

## Conflict of Interest

None declared.
